# Mitochondrial permeability transition regulator, cyclophilin D, is transcriptionally activated by C/EBP during adipogenesis

**DOI:** 10.1016/j.jbc.2023.105458

**Published:** 2023-11-08

**Authors:** Chen Yu, Rubens Sautchuk, John Martinez, Roman A. Eliseev

**Affiliations:** 1 Center for Musculoskeletal Research, University of Rochester, Rochester, New York, USA; 2 Department of Pathology, University of Rochester, Rochester, New York, USA; 3 Department of Biology, University of Rochester, Rochester, New York, USA; 4 Department of Pharmacology & Physiology, University of Rochester, Rochester, New York, USA

**Keywords:** adipogenesis, cyclophilin D, mitochondrial permeability transition, C/EBP, mesenchymal stem cells

## Abstract

Age-related bone loss is associated with decreased bone formation, increased bone resorption, and accumulation of bone marrow fat. During aging, differentiation potential of bone marrow stromal (a.k.a. mesenchymal stem) cells (BMSCs) is shifted toward an adipogenic lineage and away from an osteogenic lineage. In aged bone tissue, we previously observed pathological opening of the mitochondrial permeability transition pore (MPTP) which leads to mitochondrial dysfunction, oxidative phosphorylation uncoupling, and cell death. Cyclophilin D (CypD) is a mitochondrial protein that facilitates opening of the MPTP. We found earlier that CypD is downregulated during osteogenesis of BMSCs leading to lower MPTP activity and, thus, protecting mitochondria from dysfunction. However, during adipogenesis, a fate alternative to osteogenesis, the regulation of mitochondrial function and CypD expression is still unclear. In this study, we observed that BMSCs have increased CypD expression and MPTP activity, activated glycolysis, and fragmented mitochondrial network during adipogenesis. Adipogenic C/EBPα acts as a transcriptional activator of expression of the CypD gene, Ppif, during this process. Inflammation-associated transcription factor NF-κB shows a synergistic effect with C/EBPα inducing *Ppif* expression. Overall, we demonstrated changes in mitochondrial morphology and function during adipogenesis. We also identified C/EBPα as a transcriptional activator of CypD. The synergistic activation of CypD by C/EBPα and the NF-κB p65 subunit during this process suggests a potential link between adipogenic signaling, inflammation, and MPTP gain-of-function, thus altering BMSC fate during aging.

Bone marrow niche is a complex microenvironment that is functionally crucial for bone maintenance and remodeling. As an essential component in the niche, marrow adipose tissue (MAT) modulates bone homeostasis and metabolism by storing energy in the form of fat and secreting various adipokines. A recent study showed that MAT potentially has distinct subpopulations termed as constitutive MAT and regulated MAT. They exhibit different cell locations, cell sizes, adipogenic gene expression patterns and responses to challenges, *e.g.*, cold exposure and aging process (1). Though a lot of questions remain unanswered in the study, it might help us better understand MAT in the future. During aging and some pathological processes including obesity and diabetes, MAT expands and accumulates in the bone marrow niche. Balance between bone formation and resorption is disrupted during the process. Several studies have revealed that MAT expansion is negatively correlated with bone mineral density increasing the risk of osteoporosis and bone fracture (1, 2, 3, 4). While the mechanisms behind these age-related changes remain unclear, it is now widely recognized that aging alters stem cell lineage commitment. Bone marrow stromal, a.k.a. mesenchymal stem, cells (BMSCs) are multipotent stem cells in the bone marrow niche. They can be induced to differentiate into osteoblasts, chondrocytes, and adipocytes. During aging, differentiation potential of BMSCs shifts from osteogenesis toward adipogenesis leading to decreased bone formation and increased MAT accumulation (5).

Mitochondria play an important role in the regulation of stem cell homeostasis and differentiation. Mitochondrial function is closely attuned to cell energy demand and metabolic requirement during cell differentiation. Several studies including ours have shown that upregulation of oxidative phosphorylation (OXPHOS) level is required during osteogenic differentiation (6, 7, 8). Thus, mitochondrial dysfunction caused by accumulated mtDNA damage, compromised mitophagy, and increased cellular stress during aging (9, 10) may lead to a glycolytic shift which impairs osteogenic differentiation potential of BMSCs.

Mitochondrial permeability transition pore (MPTP) is a nonselective high conductance pore in the inner mitochondrial membrane. Though the identity of MPTP is still under debate, it has been reported that F_O_F_1_-ATP synthase is one of the essential components of MPTP (11, 12). Transient opening of MPTP is associated with calcium homeostasis (13, 14, 15), while sustained opening leads to mitochondrial swelling, dissipation of mitochondrial membrane potential, and eventually cell death. Cyclophilin D (CypD) is a highly conserved peptidyl-prolyl *cis*-*trans* isomerase in the mitochondrial matrix. It is the only genetically proven important regulator of MPTP which promotes opening of the pore to stimuli, such as excess calcium and reactive oxygen species (ROS). Also, CypD serves as a scaffold protein by bringing various proteins together to affect signaling pathways. So far, several studies including ours have reported that MPTP activity and CypD expression are downregulated during cell differentiation including neuronal differentiation (16), cardiomyocyte differentiation (17, 18), and osteogenic differentiation (16, 19). As these lineages are energy-demanding and require higher mitochondrial function, cells need to downregulate MPTP activity and CypD levels to increase mitochondrial membrane integrity and potential.

CypD is encoded by a nuclear gene named *Ppif*. CypD expression is controlled by many different posttranslational modifications such as phosphorylation, acetylation, and S-nitrosation (20, 21). In contrast, there are few studies on transcriptional regulation of *Ppif* gene. Previously, our lab has reported that Smad1, a mediator of BMP signaling pathway, is a transcriptional repressor of *Ppif* leading to downregulation of CypD during osteogenic differentiation (19). As an alternative fate for BMSCs, adipogenesis is a highly orchestrated process that involves many different transcription factors (TFs), *e.g.*, PPARγ and some C/EBP family members (22). However, it is still unclear whether and how CypD is regulated during this process. In this study, we explore CypD expression and mitochondrial morphology during adipogenesis in BMSCs and demonstrate transcriptional regulation of CypD by C/EBPα and NF-κB p65 during the process. Our data suggest a potential link between CypD expression and age-related changes in BMSCs during aging.

## Results

### CypD expression and MPTP activity are upregulated during adipogenesis

**Figure 1:**
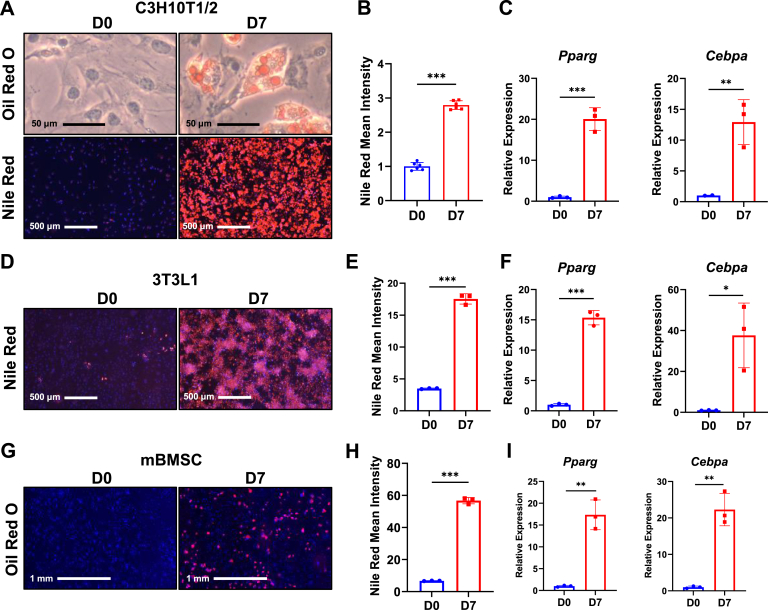
**Adipogenic induction of C3H10T1/2, 3T3L1, and primary mouse bone marrow stromal cells (mBMSCs).** C3H/10T1/2 cells, 3T3L1 cells, and primary mBMSCs were cultured in adipogenic media for 7 days. *A*, C3H10T1/2 cells at D0 and D7 were stained with Oil Red O or Nile Red/Hoechst. *B*, quantification of Nile Red staining. *C*, real-time RT-PCR analysis of adipogenic gene expression in C3H10T1/2 cells were normalized to *B2m*. *D*, 3T3L1 cells at D0 and D7 were stained with Nile Red/Hoechst. *E*, quantification of Nile Red staining. *F*, real-Time RT-PCR analysis of *Cebpa* in 3T3L1 cells were normalized to *B2m*. *G*, C57BL/6J primary mBMSCs from femur and tibia were stained with Nile Red at D0 and D7. *H*, quantification of Nile Red staining. *I*, real-time RT-PCR analysis of adipogenic gene expression in mBMSCs were normalized to *B2m*. Data are mean ± SD (n = 3), unpaired *t* test. ∗*p* < 0.05; ∗∗*p* < 0.01; ∗∗∗*p* < 0.001.

To study CypD/MPTP potential changes during adipogenesis, we used C3H10T1/2 cells, a mouse mesenchymal cell line and an established model for adipocyte differentiation (23, 24, 25). Cells were incubated in adipogenic media for 7 days. Oil Red O and Nile Red staining were performed at day 0 and day 7 and showed lipid droplet accumulation in cytoplasm at day 7 (Fig. 1, *A* and *B*). To further confirm adipogenesis, we performed real-time RT-PCR analysis and observed significant increases of adipogenic marker genes, *Pparg* and *Cebpa*, at day 7 (Fig. 1*C*). To exclude the possibility that the effect was cell line-specific, we also used 3T3-L1 cells, a mouse embryonic preadipocyte cell line and another common *in vitro* model for adipocyte studies (26). Similar to the C3H10T1/2 cells, 3T3-L1 cells accumulated lipid droplets (Fig. 1, *D* and *E*) and upregulated adipogenic gene expression (Fig. 1*F*) after 7-days adipogenic induction. As our main interest is bone marrow adipocytes, we isolated and cultured mouse BMSCs (mBMSCs) from tibiae of C57BL/6J mice. After 7-days adipogenic induction, Nile Red staining showed lipid droplet formation in mBMSCs (Fig. 1, *G* and *H*). Expression level of adipogenic marker genes was also increased at day 7 (Fig. 1*I*).

**Figure 2:**
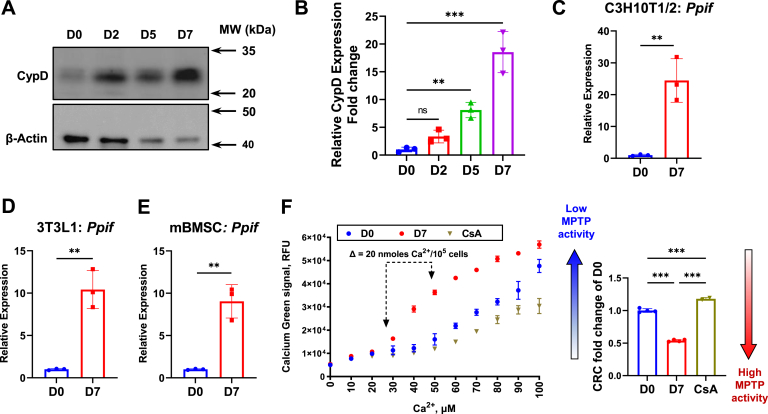
**CypD expression and MPTP activity are upregulated during adipogenesis.** C3H/10T1/2 cells, 3T3L1 cells, and primary mBMSCs were cultured in adipogenic media for 7 days. *A*, representative Western blot image of CypD protein expression. *B*, quantification of Western blot analysis. *C*, real-Time RT-PCR analysis of *Ppif* in C3H10T1/2 cells were normalized to *B2m. D*, real-Time RT-PCR analysis of *Ppif* in 3T3L1 cells were normalized to *B2m. E*, real-Time RT-PCR analysis of *Ppif* in mBMSCs were normalized to *B2m*. *F*, calcium retention capacity (CRC) assay measures calcium uptake by mitochondria at D0, D7, and D0 treated with CsA, a CypD inhibitor, in C3H10T1/2 cells (*Left*). CRC was calculated and plotted as fold change of D0 (*Right*). Data are mean ± SD (n = 3), unpaired *t* test, one-way ANOVA with post-hoc Dunnett or Tukey test. ∗∗*p* < 0.01; ∗∗∗*p* < 0.001. BMSC, bone marrow stromal cells; CypD, cyclophilin D; mBMSC, mouse BMSC; MPTP, mitochondrial permeability transition pore.

During adipogenic differentiation, we found that CypD protein expression in C3H10T1/2 cells (Fig. 2, *A* and *B*) and mBMSCs (Fig. S1, *D* and *E*) was increased. *Ppif* mRNA expression was also significantly upregulated (Fig. 2*C*). Since some reports indicated the role of mitochondrial biogenesis in adipogenesis (27), we investigated whether these changes were a reflection of overall increase in mitochondrial copy numbers. Copy numbers of mitochondrial gene (*mt-Co3*) and nuclear *18S* gene were detected by qPCR analysis. While an increase in mtDNA copy number (Fig. S1*A*) during adipogenesis was only 2-fold, *Ppif* mRNA was increased 20-fold (Fig. 2*C*), indicating that *Ppif* upregulation is only partly due to the increased mitochondrial content. Similarly, *Ppif* upregulation was confirmed in 3T3L1 preadipocytes (Fig. 2*D*) and mBMSCs (Fig. 2*E*). In differentiated 3T3L1 cells, we observed a nonsignificant increase in mtDNA copy number (Fig. S1*B*). We also analyzed public RNA-seq data and found that adipocytes were reported to have higher *Ppif* expression in comparison to mesenchymal stem cells (Fig. S1*C*).

To find out if the observed upregulation of CypD during adipogenesis coincides with increased MPTP activity, we used calcium retention capacity (CRC) assay (28, 29). As important regulators of calcium homeostasis, mitochondria take up cytosolic calcium until mitochondrial calcium overload triggers opening of MPTP (28, 30). Therefore, mitochondrial capacity of calcium uptake and retention is in reverse relationship to MPTP activity and is used as a measure of MPTP activity in the CRC assay. When reaching maximum CRC, mitochondria will release calcium through MPTP showing a sudden increase of calcium indicator signal. As a control, we showed that C3H10T1/2 cells have increased CRC when treated with cyclosporin A, a proven CypD/MPTP inhibitor. After 7 days of adipogenic induction, CRC was significantly lower when compared to undifferentiated cells (Fig. 2*F*). This finding suggests that MPTP activity is increased in adipocytes, which is consistent with the increased CypD expression. Overall, these data indicate that CypD expression and MPTP activity are upregulated during adipogenesis.

### Mitochondrial morphology during adipogenesis

**Figure 3:**
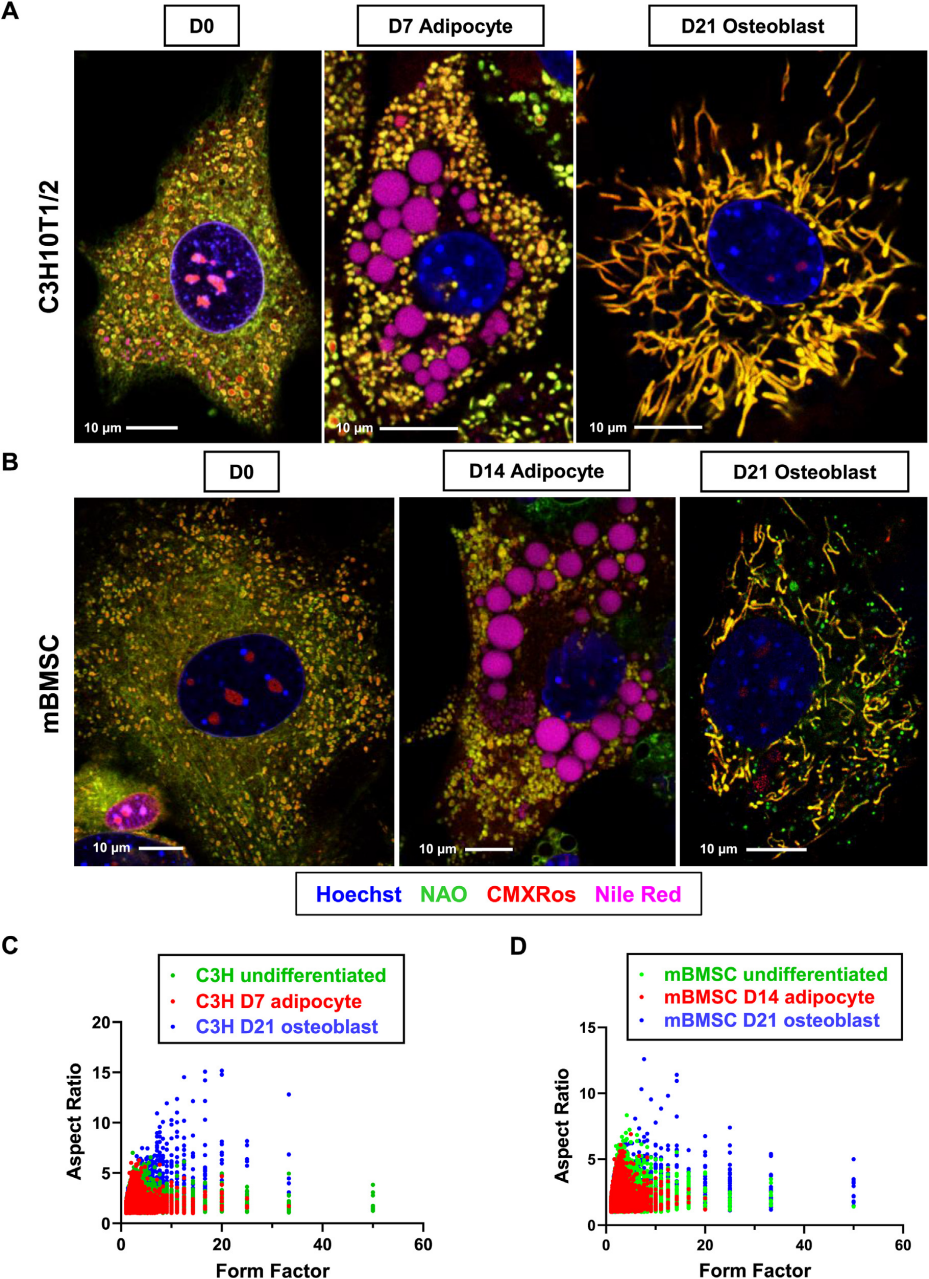
**Mitochondrial network during osteogenesis and adipogenesis in C3H10T1/2 and mBMSCs.***A*, representative images of mitochondrial morphology in C3H/10T1/2 cells. Cells were cultured in adipogenic media for 7 days or osteogenic media for 21 days. *B*, representative images of mitochondrial morphology in primary mouse BMSCs. Cells were isolated from tibia and femur and cultured in adipogenic media for 14 days or osteogenic media for 21 days. After staining, images were taken by confocal microscopy at 63× magnification. *C* and *D*, quantification of mitochondrial morphology. For each group, three cells were randomly selected for analysis. BMSC, bone marrow stromal cells; mBMSC, mouse BMSC.

Mitochondria are highly dynamic organelles that play important roles in cell division, cell differentiation, and cell death (31). By the process of fusion and fission, mitochondrial morphology is changed in response to cell fate and energy demand. Mitochondrial fission often occurs during cell proliferation whereas mitochondrial fusion is required for cells that rely on OXPHOS (32, 33). Several studies, including ours, have shown that BMSCs undergo mitochondrial fusion during osteogenic differentiation (6, 7, 8). In the current study, we also observed an increase in CypD level and MPTP activity during adipogenesis. MPTP opening leads to mitochondrial swelling and rounding which can be visualized using confocal or electron microscopy (6). Therefore, we investigated whether mitochondrial morphology was altered during adipogenesis. CMXRos is a red fluorescent dye that accumulates preferably in the mitochondrial matrix. Nonyl acridine orange (NAO) is a green, fluorescent dye that labels cardiolipin found mostly in the inner mitochondrial membrane. We used these dyes to assess mitochondrial morphology in high-resolution confocal microscope. C3H10T1/2 cells and mBMSCs were incubated in either osteogenic media or adipogenic media. Cells were then stained with CMXRos, NAO, nuclear staining Hoechst33342, and Nile Red (undifferentiated cells and adipocytes only). In undifferentiated C3H10T1/3 cells and mBMSCs, mitochondria appeared rounded and fragmented (Fig. 3, *A* and *B*). During osteogenic differentiation, mitochondria became elongated forming an interconnected network (Fig. 3, *A* and *B*), consistent with previous reports by us and others (34, 35). However, during adipogenic differentiation, mitochondria remain rounded and fragmented. Furthermore, they were swollen when compared with osteoinduced cells (Fig. 3, *A* and *B*). To analyze mitochondrial morphology, form factor (FF) and aspect ratio (AR) were calculated to describe mitochondrial branching and shape, respectively. CMXRos channel was used for analysis (Fig. S2). Images were processed and analyzed using particle analysis in ImageJ (Fig. S3). Quantification of mitochondrial morphology shows that osteoblasts have larger AR and FF value, suggesting highly branched mitochondria and complexed networks (Fig. 3, *C* and *D*). In contrast, undifferentiated cells and adipocytes show similar distribution of each parameter, indicating round mitochondrial shape and fragmented networks (Fig. 3, *C* and *D*). Taken together, our data demonstrate that in contrast to osteogenesis, mitochondria remain rounded, fragmented, and even partially swollen during adipogenesis.

### Glycolysis is significantly upregulated during adipogenesis in C3H10T1/2 cells

**Figure 4:**
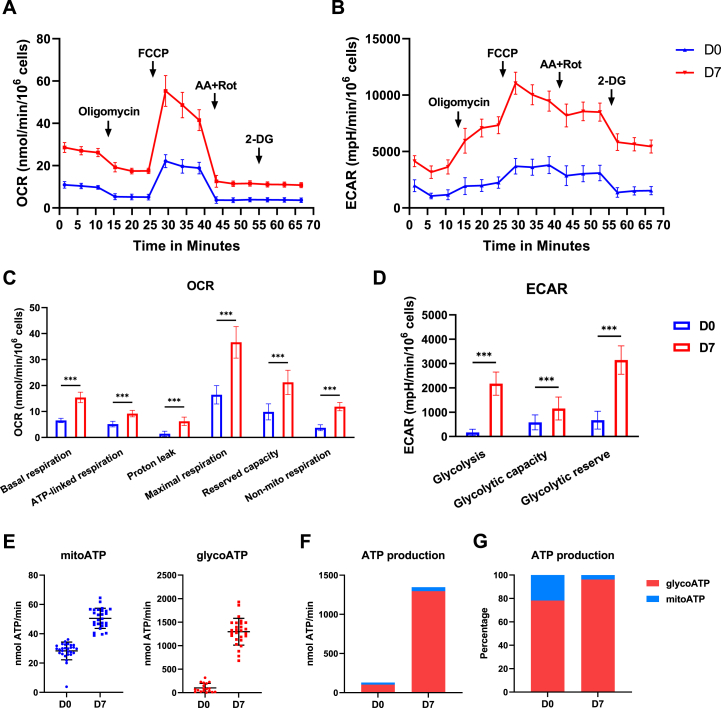
**C3H10T1/2 cells greatly increase glycolysis level during adipogenesis.** C3H10T1/2 cells were cultured in Seahorse XFe96 plates and incubated in adipogenic media for 7 days. Oxygen consumption rate (OCR) and extracellular acidification rate (ECAR) were measured in an Agilent Seahorse XFe96 Analyzer. OCR and ECAR levels were normalized to cell number assessed by Hoechst staining. *A* and *B*, kinetic profile of OCR and ECAR, respectively. *C* and *D*, quantification of OCR and ECAR data, respectively. *E*, quantification of mitochondrial and glycolysis ATP production rate. *F*, total ATP production rate. *G*, percentage of ATP production rate. Data are mean ± SD (n = 28), unpaired *t* test. ∗∗∗*p* < 0.001.

Given that MPTP activity is upregulated and mitochondria are fragmented during adipogenesis, we anticipated bioenergetic changes in adipogenesis and therefore evaluated OXPHOS and glycolysis level using Seahorse XFe96 Analyzer. Previously, we reported activation of OXPHOS with insignificant changes in glycolysis during osteogenic differentiation (6, 35). In the current study, we monitored cellular oxygen consumption rate (OCR), an OXPHOS measure, and extracellular acidification rate (ECAR), glycolysis measure, in undifferentiated C3H10T1/2 cells and adipocytes over time in response to multiple drugs (Fig. 4, *A* and *B*). The assay showed that basal respiration, ATP-linked respiration, proton leak, maximum respiration, and reserved capacity were increased by 2-fold in adipocytes when compared to undifferentiated cells (Fig. 4*C*). On the other hand, glycolytic capacity and reserve were upregulated by 2- and 5-fold (Fig. 4*D*), respectively. Basal glycolysis was increased by 20-fold in adipocytes (Fig. 4*D*), suggesting a metabolic shift toward glycolysis during adipogenic differentiation. To determine the proportions of ATP generated from OXPHOS and glycolysis, ATP production rates were calculated using OCR and ECAR data (Fig. 4*E*). We found that total ATP production was increased greatly at day 7 of adipogenic induction with majority of the ATP generated from glycolysis in both undifferentiated cells and adipocytes (Fig. 4*F*). Though mitochondrial ATP production was increased, its percentage in total ATP production was decreased in adipocytes *versus* undifferentiated cells (Fig. 4*G*). These data demonstrate that adipogenesis of mesenchymal lineage cells induces a metabolic shift with pronounced upregulation of glycolysis.

Ascorbate is critical for osteogenic differentiation. It stimulates collagen synthesis and bone mineralization. Furthermore, ascorbate is an antioxidant to protect cells from a variety of ROS during differentiation. To explore the impact of ascorbate on adipogenesis, we treated C3H10T1/2 cells during adipogenic differentiation at D4 or after D7 and D10 of adipogenic induction. At D7 and D10, we did not find significant differences in lipid droplet accumulation as well as ROS level between control and treatment group (Fig. S5), suggesting that 50 μg/ml ascorbate does not affect adipogenesis of BMSCs.

### C/EBPα transcriptionally regulates *Ppif* gene expression

**Figure 5:**
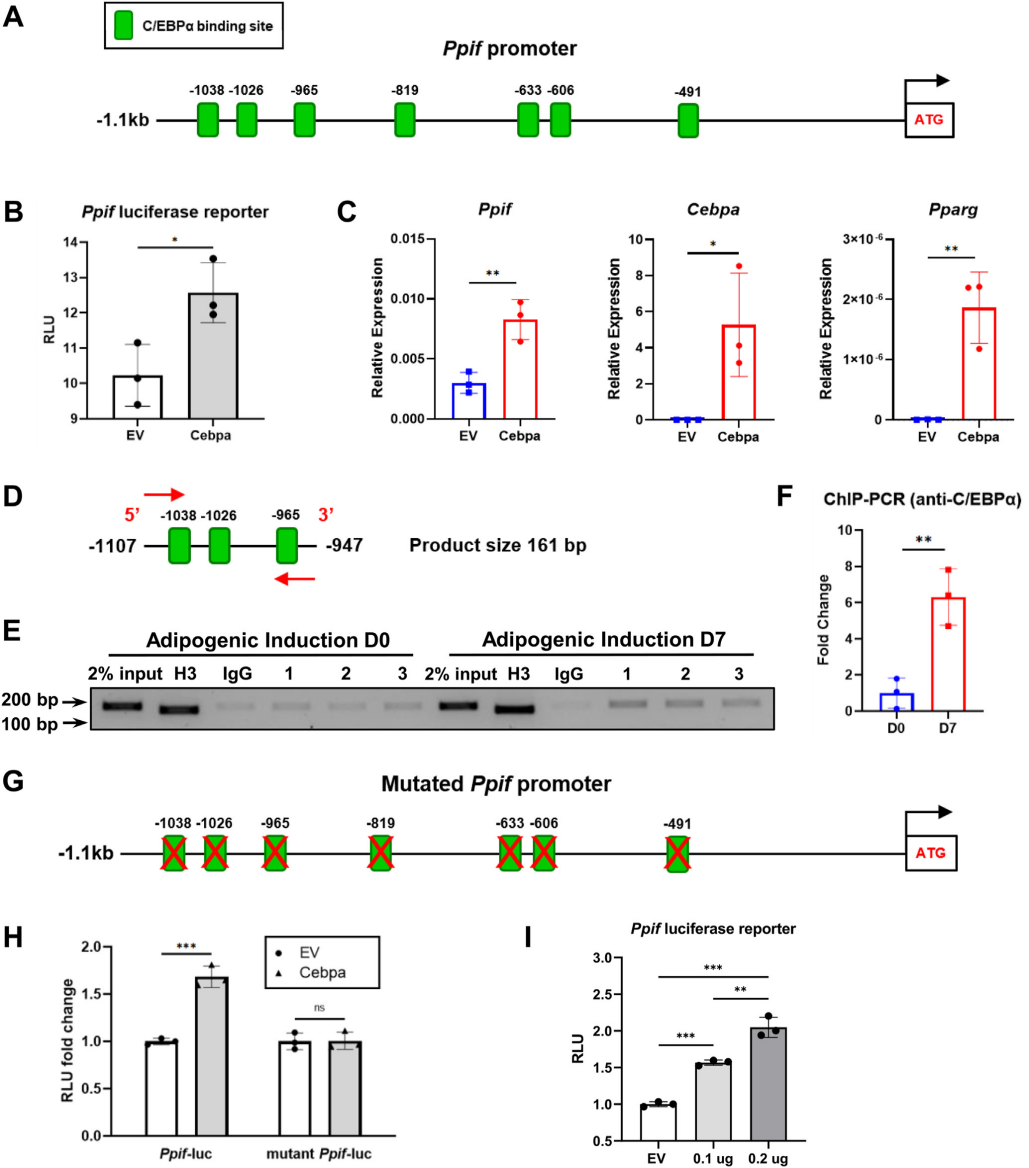
**C/EBPα binds *Ppif* promoter and activates transcriptional activity of *Ppif* promoter luciferase reporter.***A*, mouse *Ppif* promoter (−1.1kb) contains multiple C/EBPα binding sites. *B*, C3H10T1/2 cells were transfected with pCMV-C/EBPα vector or empty vector (EV) as control. *C*, real-time RT-PCR analysis was normalized to *B2m*. *Cebpa* and *Pparg* expression confirms *Cebpa* overexpression. *D*, primer for ChIP-PCR analysis amplifies distal region of *Ppif* promoter containing several C/EBPα binding sites. *E*, ChIP-PCR analysis of C3H10T1/2 cells at D0 and D7. Histone H3 and IgG are positive and negative control, respectively. *F*, quantification of PCR analysis. *G*, mutated mouse *Ppif* promoter (−1.1kb) with scrambled C/EBPα binding sites. *H*, C3H10T1/2 cells were transfected with wildtype or mutant *Ppif* luciferase reporter. Results was normalized to data of pGL4.10 transfection. *I*, C3H10T1/2 cells were transfected with different doses of pCMV-C/EBPα. Data are mean ± SD (n = 3), unpaired *t* test, or one-way ANOVA with post-hoc Tukey test. ∗*p* < 0.05; ∗∗*p* < 0.01; ∗∗∗*p* < 0.001. ChIP, chromatin immunoprecipitation assay.

Our data showing that *Ppif* mRNA levels were induced during adipogenesis indicate that it was regulated on a transcriptional level. To determine potential mechanisms of *Ppif* transcriptional regulation during adipogenesis, we performed TF binding site prediction analysis of the promotor region of *Ppif* gene using PROMO web-based tool (36). Analysis of the 1.1 kb upstream region of *Ppif* promoter revealed multiple potential binding sites for C/EBPα TF (Figs. 5*A* and S6*C*). As a master regulator of adipogenesis, C/EBPα is expressed at high levels during terminal adipogenic differentiation. C/EBPα and PPARγ, another key regulator of adipogenesis, can activate each other’s expression to maintain differentiated state (37, 38). To confirm the involvement of C/EBPα in regulation of *Ppif* expression, we performed luciferase reporter assay. C3H10T1/2 cells were cotransfected with pCMV-C/EBPα or pCMV empty vector (EV) and *Ppif-luc* promoter-reporter construct as previously described (19). Transfection of pCMV-C/EBPα showed significantly higher luciferase signal when compared to EV, indicating its potential role as *Ppif* transcriptional activator (Fig. 5*B*). Transfection efficiency was confirmed by real-time RT-PCR analysis of *Pparg* and also of *Cebpa* a target gene of C/EBPα (Fig. 5*C*). Interestingly, *Ppif* mRNA level (Fig. 5*C*) and protein level (Fig. S6, *A* and *B*) were also upregulated after transfection, confirming C/EBPα as a transcriptional activator of *Ppif* expression.

To confirm the binding of C/EBPα to *Ppif* promoter region, we performed chromatin immunoprecipitation assay (ChIP) using nuclear lysates collected from undifferentiated C3H10T1/2 cells and adipogenically induced cells. A primer pair amplifying the distal region of *Ppif* promoter was used for PCR analysis (Fig. 5*D*). We found that the interaction between C/EBPα and *Ppif* promoter remained at a low level in undifferentiated cells. However, the binding activity was significantly increased after 7-days adipogenic induction (Fig. 5, *E* and *F*). To confirm the specificity of the interaction, we created a mutant *Ppif* promoter luciferase reporter containing mutated binding sequences (Figs. 5*G* and S6*D*). Compared to the wildtype *Ppif-luc* promoter-reporter, mutant reporter showed significantly reduced luciferase signal in response to pCMV-C/EBPα transfection, not different from the one in response to the EV transfection. Thus, mutation of predicted C/EBPα binding sites abolished the transcriptional activation effect on *Ppif* promoter-reporter (Fig. 5*H*). To determine if *Ppif* promoter activity depends on C/EBPα concentration, we used different doses of pCMV-C/EBPα to transfect C3H10T1/2 cells. The data indicate that the response of *Ppif* promoter to C/EBPα is dose-dependent and positively associated with C/EBPα concentration (Fig. 5*I*). Taken together, these data indicate that C/EBPα act as a transcriptional activator of *Ppif* gene during adipogenesis.

### NF-κB p65 and C/EBPα synergistically regulate *Ppif* gene expression

**Figure 6:**
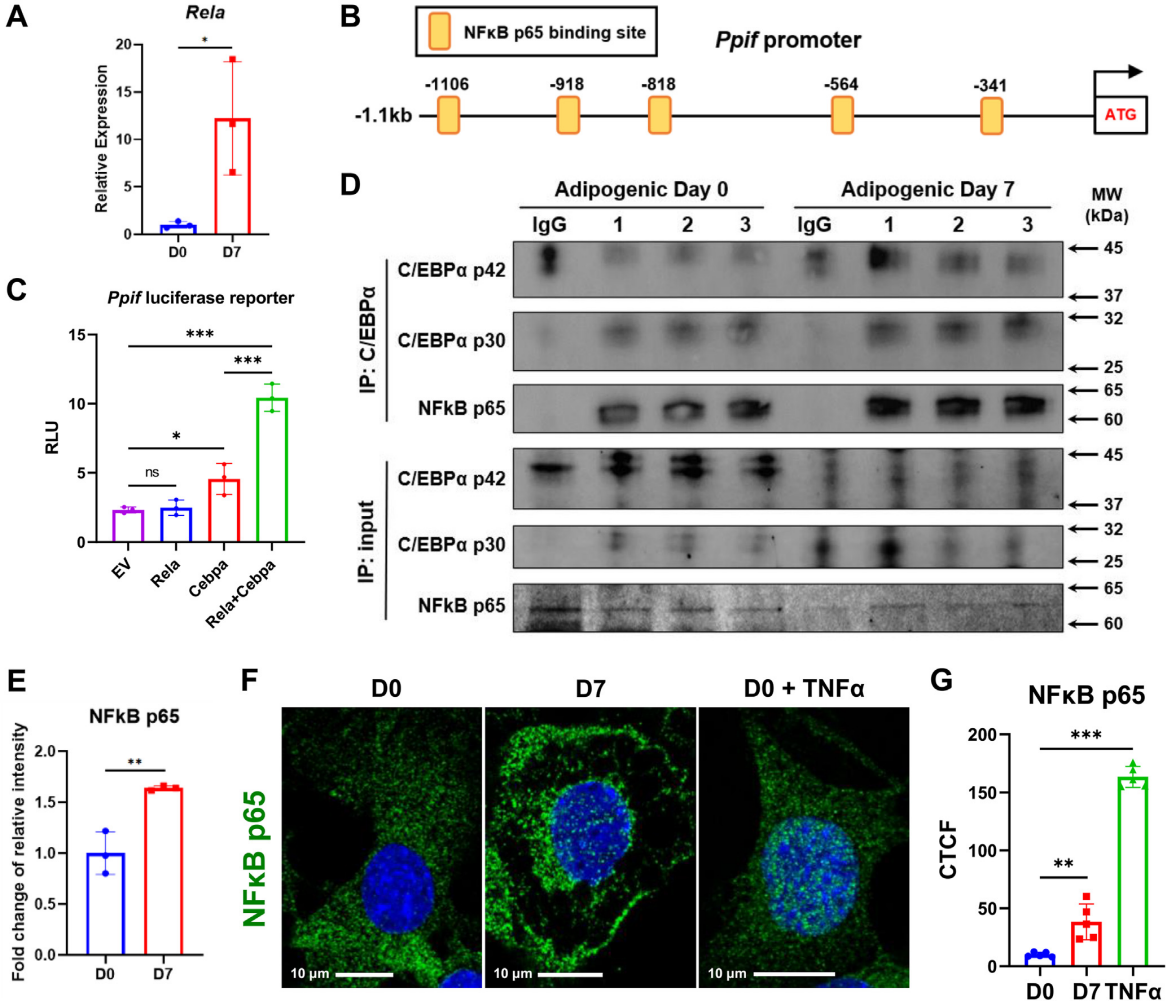
**Synergistic effect of NF-κB p65 and C/EBPα on *Ppif* gene during adipogenesis.***A*, mouse *Ppif* promoter (−1.1kb) contains multiple NF-κB p65 binding sites. *B*, real-time RT-PCR analysis of *Rela* gene. *C*, C3H10T1/2 cells were transfected with pCMV-p65, pCMV-C/EBPα vector or empty vector (EV) as control. *D*, cell lysates were collected from undifferentiated C3H10T1/2 cells and day 7 adipocytes. Co-immunoprecipitation (co-IP) was performed with anti-C/EBPα, anti-p65, or nonspecific rabbit immunoglobulins as negative control. *E*, quantification of NF-κB p65 bands in co-IP. Data were normalized to IP C/EBPα p30 expression. *F*, C3H10 T/12 cells were treated with 20 ng/ml TNFα for 30 min as positive control. Samples were fixed and stained with anti-p65 primary antibody and Alexa Fluor 488-conjugated IgG antibody. Images were taken by confocal microscopy at 63x magnification. *G*, quantification of fluorescence signal. Data are mean ± SD (n = 5 in G, n = 3 for others), unpaired *t* test, one-way ANOVA with post-hoc Dunnett or Tukey test. ∗*p* < 0.05; ∗∗*p* < 0.01; ∗∗∗*p* < 0.001.

Aging is a major factor in many bone disorders including aging-related osteoporosis and osteoporotic fracture. During aging, decreased osteogenic potential and increased adipogenic potential of BMSCs contribute to bone loss and fat accumulation (39). Chronic inflammation in various tissues is considered as a major factor of many aging-associated pathologic conditions including age-related bone loss (40). Both extrinsic proinflammatory environment and cell-intrinsic inflammatory signaling lead to the dysregulated differentiation of BMSCs. NF-κB signaling is a key regulator of inflammatory responses and is also involved in controlling cell differentiation and activity of osteoblast, osteocyte, osteoclast, chondrocyte, and adipocyte (41). Several studies have shown that NF-κB activation inhibits osteogenesis and osteoblast function (42, 43, 44, 45), implicating a role of NF-κB signaling in regulating BMSC differentiation during aging. NF-κB is a family of five members: RelA (p65), p50, p52, RelB, and c-Rel. In canonical pathway, RelA/p65 preferentially heterodimerizes with p50 forming the most prevalent dimer in response to many factors including RANKL, TNF, and IL-1 (46). It has been shown that RelA/p65 and RelB are upregulated during adipogenesis (47). Consistent with these data, we found that C3H10T1/2 cells upregulated *Rela* expression after 7-days adipogenic induction (Fig. 6*A*. Therefore, we examined whether p65 is involved in the transcriptional regulation of *Ppif* gene. Our PROMO analysis showed that *Ppif* gene promoter contains several NF-κB p65 binding sites (Fig. 6*B*). *Ppif* promoter-reporter assay showed that p65 alone does not induce luciferase activity but synergistically activates *Ppif* gene transcription with C/EBPα (Fig. 6*C*). To determine possible interaction of p65 and C/EBPα, during regulation of *Ppif* expression, we performed co-immunoprecipitation assay in undifferentiated cells or adipocytes. Protein lysates were incubated with anti-C/EBPα primary antibody. In IgG negative control, because mouse IgG heavy chain has similar molecular weight with C/EBPα p42 isoform (Fig. 6*D*), we used C/EBPα p30 expression for quantification analysis. Western blot showed that p65 and C/EBPα have increased binding activity in adipocytes when compared to undifferentiated cells (Fig. 6, *D* and *E*). To confirm activation of NF-κB signaling, we used immunofluorescence to detect nuclear translocation of p65. C3H10T1/2 cells were also treated with 20 ng/ml TNFα for 30 min as a positive control. Immunostaining with anti-p65 antibody showed that adipogenic differentiation induces p65 nuclear translocation after 7 days (Fig. 6, *F* and *G*), suggesting a potential role of NF-κB p65 in transcriptional regulation. Taken together, these data demonstrate that during adipogenesis, NF-κB p65 translocates to the nucleus and interacts with C/EBPα to potentially enhance *Ppif* gene transcription activity.

## Discussion

Mitochondrial metabolism is closely linked to stem cell proliferation and differentiation. To fulfill the energy demand, mitochondria utilize the electron transport chain (ETC) to produce ATP. To support anabolic requirement, metabolites in TCA cycle such as citrate, oxaloacetate and α-ketoglutarate are used to generate macromolecules including fatty acids, amino acids and nucleotides. In addition, mitochondria have the potential to control stem cell fate. Some TCA cycle substrates are essential for regulation of epigenetic reactions. Acetyl-coenzyme A provides the acetyl group for histone acetylation. α-Ketoglutarate is a cofactor of JMJD histone demethylases and TET enzymes that mediate DNA methylation (48). Mitochondrial NAD^+^/NADH ratio is critical for the enzymatic activity of sirtuins, a family of NAD^+^-dependent protein deacylases, and therefore regulate stem cell fate (49, 50, 51). ETC and cytosolic NADPH oxidases are two major sources of ROS. A basal low level of ROS is essential to maintain stemness, whereas a slight increase of ROS is necessary for cell differentiation of induced pluripotent stem cells and embryonic stem cells (52). During cell differentiation, stem cells exit the quiescent state and transform into a different cellular and metabolic state. In fact, as we mentioned above, mitochondrial activation has been observed during differentiation of several different cell types including neurons (16, 53), cardiomyocytes (17, 18, 54), and osteoblasts (6, 7, 8, 35). This shift from glycolysis to OXPHOS accommodates BMSCs to meet the new metabolic needs during osteogenic differentiation.

The lineage commitment of BMSCs toward osteogenesis *versus* adipogenesis has been extensively studied for the past few decades. Similar to the negative correlation between MAT and bone mineral density, *in vitro* studies have demonstrated an inverse relationship between osteogenesis and adipogenesis (55, 56). Previously, our lab has reported that glycolysis level is maintained, while mitochondrial function is upregulated, and CypD expression is downregulated during osteogenic differentiation of BMSCs (6, 19, 35). In this study, we also show that C3H10T1/2 mesenchymal cells increase OXPHOS but maintain glycolysis level during osteogenic differentiation (Fig. S4). As an alternative and opposite cell fate, adipogenesis shows increased CypD expression in C3H10T1/2 mesenchymal cells, 3T3L1 preadipocytes, and mouse primary BMSCs. Consistent with this finding, we observed higher MPTP activity and fragmented and rounded mitochondria in adipocytes. Since the pathological prolonged opening of MPTP results in OXPHOS uncoupling, mitochondrial swelling and eventually cell death (57), we speculate that MPTP opening in adipocytes is transient and fine-tuned. This phenomenon has been implicated before in the regulation of ROS signaling and Ca^2+^ overload in cardiomyocytes, in which most of the mitochondria are still functional, while some mitochondria have decreased membrane potential temporarily (58, 59). ROS level is increased during adipogenic differentiation and is necessary for this cell fate determination (60, 61). In contrast, excessive ROS impairs adipogenesis and causes adipose tissue dysfunction (62). Several studies have shown that adipogenesis is accompanied by upregulation of mitochondrial gene and protein level (27, 63, 64, 65, 66). In this study, we also show that mitochondrial parameters obtained from OCR measurement are upregulated by 2-fold in adipocytes. Since ETC is one of the major sources of ROS, it is possible that this well-regulated MPTP opening helps maintain redox homeostasis in adipocytes. On the other hand, though some studies have claimed that BMSCs undergo a shift from glycolysis to OXPHOS during adipogenic differentiation (67, 68), we observed a 20-fold increase of basal glycolysis level during adipogenic differentiation. Calculation of ATP production reveals that ATP production is significantly increased after adipogenic differentiation, while the vast majority of ATP is produced from glycolysis in both undifferentiated cells and adipocytes, suggesting that adipocytes undergo a metabolic change relying on glycolysis rather than OXPHOS. It is consistent with the fact the CypD expression and MPTP opening are increased in adipocytes. It is also possible that high CypD expression and MPTP activity not only put a brake on the mitochondrial OXPHOS function but also increase the availability of TCA cycle substrates, such as citrate, in the cytosol. This is required in order to meet the new metabolic demands of the cell including the active fatty acid synthesis in adipocytes.

Adipogenesis is an orchestrated process that involves a variety of transcriptional factors and signaling pathways. The C/EBP family is consist of at least seven members that are important for cell differentiation and proliferation. These TFs contain a C-terminal leucine zipper, a DNA-binding region, and a N-terminal transactivating region (69). They can form either homodimer or heterodimer with each other *via* the leucine zipper domain. In the early phase of adipogenesis, C/EBPβ and C/EBPδ are induced in response to cyclic AMP signaling. Expression of C/EBPβ in turn activates PPARγ. In the late stage, PPARγ and C/EBPα, the two master regulators of adipogenesis, transactivate each other forming a feedback loop to promote and maintain adipogenic differentiation (22). C/EBPα mRNA gives rise to two isoforms p42 and p30 as a result of alternative translation initiation. Compared with the larger isoform, C/EBPα p30 has fewer N-terminal transactivation domains and hence a lower transcriptional activation potential (70, 71). Analysis of the 5′ upstream 1.1kb *Ppif* promoter region reveals multiple C/EBPα binding sites. Transfection of pCMV-C/EBPα (p42) plasmid induces both *Ppif* luciferase reporter and *Ppif* mRNA expression. Furthermore, this transcriptional activation effect on *Ppif* luciferase reporter expression is dose-dependent, suggesting a potential positive correlation between C/EBPα-mediated adipogenesis and CypD expression level. As we expected, the physical interaction between C/EBPα protein and *Ppif* promoter region is confirmed by ChIP-PCR assay. This interaction is further validated by the fact that mutation of each predicted C/EBP binding site on the *Ppif* promoter abolishes *Ppif* promoter reporter activation by C/EBPα. C/EBPα p42 has been shown to inhibit cell proliferation and induce transcription of adipogenic genes such as *Fabp4*. In contrast, C/EBPα p30 is insufficient to inhibit cell proliferation and induce adipogenic differentiation. However, the ratio of p42 and p30 changes during adipogenic differentiation of 3T3-L1 cells with a peak at day 4 and 5 (71). Interestingly, we also found C/EBPα p42 and p30 are differentially expressed in undifferentiated cells and mature adipocytes, implicating a potential role of C/EBPα p42/p30 ratio during adipogenic differentiation.

Aging and age-related diseases are associated with chronic inflammation within tissues and increased intracellular inflammatory signaling. NF-κB family members are important regulators of inflammatory responses (72). During aging, several pathways are activated and finally converge in the regulation of NF-κB signaling pathway, such as pro-survival insulin/IGF-1 signaling, mTOR signaling, and DNA damage response pathway (73, 74, 75). Notably, we found multiple NF-κB binding sites in the 1.1kb *Ppif* promoter region. TFs in NF-κB family play critical roles in inflammation, cell proliferation, cell survival and cell differentiation. Once activated, NF-κB dimers in cytosol are released from the inhibitor of NF-κB proteins (IκBs) and translocate into the nucleus to regulate downstream genes. Similar with C/EBP family, NF-κB family members can form homo- or heterodimers with each other. NF-κB p65/p50 heterodimer is the most abundant form mediating the canonical NF-κB pathway (76). In this study, we demonstrate that expression of NF-κB p65 is upregulated during adipogenic differentiation. Nuclear translocation of NF-κB p65 is also observed during this process, suggesting activation of NF-κB signaling pathway in adipocytes. Previously it has been reported that NF-κB family functionally synergizes with C/EBP family by forming heterodimers (77). In fact, transfection of NF-κB p65 vector alone has no effect on *Ppif* luciferase reporter whereas co-transfection of vectors expressing NF-κB p65 and C/EBPα p42 shows synergistic effect on the reporter. The interaction between NF-κB p65 and C/EBPα is confirmed by co-immunoprecipitation. During aging, BMSCs have shortened telomeres and accumulate DNA damage and ROS, leading to increased NF-κB signaling, which eventually decreases proliferative capacity and impairs differentiation potential. It has been shown that activation of NF-κB signaling impairs osteogenesis of BMSCs (44, 78). Meanwhile, chronic inflammation is developed in the aged bone marrow microenvironment in the absence of infection resulting in alteration of bone marrow composition (79). These intrinsic and extrinsic ‘inflamm-aging’ changes potentially contribute to an overactive NF-κB signaling in BMSCs which leads to increased CypD expression, higher MPTP activity, decreased mitochondrial function and therefore, impaired osteogenic potential and increased adipogenic potential.

In summary, we found that CypD and MPTP activity are upregulated during adipogenic differentiation. To meet the metabolic demands of adipogenesis, glycolysis level is significantly increased in adipocytes. It is consistent with the observation of fragmented, rounded, and even partially swollen mitochondria in mature adipocytes. Transcriptional regulation of *Ppif* expression by C/EBPα and NF-κB p65 suggests a potential mechanism for age-related change of differentiation capacity in BMSCs. As a future direction, we are currently pursuing the effect of CypD gain- and loss-of-function in BMSCs *in vitro* and *in vivo* aiming to elucidate the role of CypD/MPTP in stem cell fate decisions and lineage allocation.

## Experimental procedures

### Isolation of BMSCs

Animal husbandry and experiments were performed upon approval of University of Rochester Institutional Animal Care and Use Committee and in accordance with state and federal law. Primary BMSCs were isolated from tibial and femoral bone marrow of 4-month-old C57BL/6J mice (Jackson Laboratory). Cells were seeded in physiologically relevant low-glucose Dulbecco's modified Eagle's medium (DMEM) (Gibco, 5 mM glucose) supplied with 10% fetal bovine serum and 1% penicillin-streptomycin at 20 × 10^6^ total bone marrow cells per 10 cm dish and incubated at 37 °C, 5% CO_2_ and 5% O_2_. Media were changed every day for 3 days to remove nonadherent cells.

### Cell culture and differentiation

C3H10T1/2 cells were purchased from ATCC. 3T3-L1 cells (ATCC) were provided by Dr Ananta Paine (University of Rochester Medical Center). Cells were expanded in low-glucose DMEM described above. For osteogenic differentiation, cells were induced with 50 μg/ml ascorbate and 2.5 mM β-glycerophosphate for 21 days. For adipogenic differentiation, cells were induced in high-glucose DMEM (Gibco, 25 mM glucose) supplied with 1 μM dexamethasone, 0.5 mM IBMX, 1 μM rosiglitazone, and 10 μg/ml insulin for 2 days and with rosiglitazone and insulin for the remaining 5 or 12 days. Media were changed every 3 days. Adipogenic differentiation was assessed by Nile Red or Oil Red O staining and adipogenic gene expression analysis. Staining data were normalized to cell number assessed *via* nuclear Hoechst 33342 staining and Celigo multiwell plate imager.

### Real-time RT-PCR

Total RNA was purified using the Qiagen RNeasy kit (74106) and reversed transcribed into cDNA using the qScript cDNA supermix (Quantabio, 95048-500). cDNA was subjected to real-time RT-PCR. The primer pairs are outlined in SI Table. Real-time RT-PCR was performed with SYBR Green (Quantabio, 95072-012) using the Qiagen RotorGene system. Reaction efficiency was calculated based on a standard curve. β2-microglobulin (*B2m*) was used as a reference gene. Raw Ct value was normalized using 2^−ΔΔCt^ method.

### Western blot

Cells were lysed in buffer containing protease inhibitors and subjected to 4 to 12% sodium dodecyl sulfate polyacrylamide gel electrophoresis followed by polyvinylidene difluoride membrane transfer. Membrane was then blocked in 5% dry milk reconstituted in PBST (PBS with 0.1% Tween 20). All antibodies were diluted in either in 2% dry milk or 1% BSA in PBST. Membrane was incubated with anti-CypD antibody (RRID: AB 478283, 1:3000), anti-beta actin antibody (RRID: AB_476697, 1:30,000), anti-C/EBPα antibody (Cell Signaling, 8178, 1:1000), anti-NF-κB p65 antibody (Cell Signaling, 8242, 1:1000), and secondary HRP conjugated goat anti-mouse or -rabbit antibody (1:3000). Signals were developed with West Femto Substrate (Thermal Fisher Scientific, 34094). We used Image Lab Software to measure band intensity and normalized CypD protein expression to β-Actin protein expression showing as fold change to D0 or control.

### CRC assay

CRC assay was performed as previously described (80). C3H10T1/2 cells (1 × 10^5^) were permeabilized with 0.01% digitonin for 3 min on ice in a KCI-based buffer (19). Permeabilization was confirmed with trypan blue staining (data not shown). Cells were then washed, resuspended, and plated in 100 μl KCI-based buffer containing 1 μM Calcium Green-5N (Invitrogen, C3737) in a 96-well black-walled and clear-bottom plate. Then, cells were exposed to pulses of Ca^2+^ (10 μM increments). Steady-state fluorescent signal after each pulse was measured using BioTek plate reader. One micrometer cyclosporin A was added to inhibit MPTP opening as a negative control. To calculate CRC, two trendlines were created before and after the Ca^2+^-releasing point, respectively. The x-value of intersection point was calculated as CRC.

### mtDNA assay

Total DNA was isolated using the Wizard SV DNA purification kit (Promega). Quantitative PCR was performed to detect the mtDNA-encoded *mt-Co3* and nuclear-encoded *18s*. The primer pairs are outlined in a Table S1.

### Mitochondrial staining and imaging

Live cells were stained with 100 nM of mitochondria-specific probe, MitoTracker Red CMXRos (Invitrogen, M7512), 100 nM of mitochondrial membrane-specific probe, NAO (Invitrogen, A1372), lipid-specific probe, Nile Red (Sigma-Aldrich, N3013), and nuclear-specific probe, Hoechst 33342 (Molecular Probes, H1339). Images were taken by Leica Stellaris 5 WLL Laser Scanning Confocal Microscopy and analyzed by ImageJ. The FF and AR are defined as:

$$Form\;Factor=\frac{{Perimeter}^{2}}{{4\pi }^{Area}}\;Aspect\;Ratio=\frac{Major\;axis\;length}{Minor\;axis\;length}$$

### Seahorse assay

OCR and ECAR were measured using Seahorse XFe96 Analyzer (Agilent). C3H10T1/2 cells were plated at a density of 20,000 cells per well in a Seahorse 96-well plate and adipogenically induced for 7 days. Immediately before the experiment, media were replaced with unbuffered DMEM media containing 1 mM L-glutamine (Gibco, 25030-08), 5 mM or 25 mM D-glucose (Sigma, G8270), and no pyruvate (pH 7.4). OCR and ECAR baseline were measured, and then an inhibitory analysis was performed with sequential injections of 4 μM oligomycin, 2 μM FCCP, 2 μM rotenone + 2 μM antimycin A, and 2 μM 2-deoxyglucose + 3 μg/ml Hoechst 33342. After analysis, total cell number was measured using Celigo cytometer based on Hoechst signal. The OXPHOS and glycolytic indexes were calculated as previously described (81).

### *Ppif* promoter luciferase reporter assay

Mouse 1.1kb *Ppif* promoter luciferase reporter was constructed as previously described (19). For mutagenesis, the 2 to 4 position of C/EBPα binding motif (predicted by PROMO web-based tool) was changed to ‘CGC’ to disrupt binding activity. The mutated promoter containing CTCGAG XhoI 5′ and AAGCTT HindIII 3′ flanking restriction sites was custom made by IDT (gBlocks Gene Fragments). The mutated promoter was then primed with XhoI and HindIII and ligated into the precut pGL4.10 luciferase reporter vector. Cells seeded at a density of 20,000 cells per well in a 12-well plate were transfected with 0.2 μg naïve or mutated *Ppif* promoter luciferase reporter and 0.2 μg pCMV-C/EBPα or empty vector (EV) control. For synergistic effect, cells were cotransfected with 0.4 μg *Ppif* promoter luciferase reporter, 0.2 μg pCMV-C/EBPα/pCMV-p65 + 0.2 μg EV, or 0.4 μg EV. The Renilla luciferase vector pRL (Promega) was cotransfected at 0.01 μg per well as a reference. Firefly and Renilla luciferase activities were assessed by an Optocomp 1 luminometer using Dual Luciferase Reporter Assay System (Promega) according to the manufacture’s protocol. The firefly luciferase signal was normalized to Renilla luciferase signal.

### ChIP-PCR assay

ChIP was performed as previously described (19). Cells were fixed and incubated with formaldehyde. Chromatin was digested, sonicated, and immunoprecipitated with either anti-C/EBPα antibody (Santa Cruz, sc-166258X), or immunoglobulin G, or histone H3 antibody using protein G magnetic beads. Fragmented chromatin was then eluted and treated with proteinase K. After purification, DNA was analyzed by PCR to amplify *Ppif* promoter distal region. Band intensities were quantified using Image Lab Software.

### Co-immunoprecipitation

Two hundred microgram of protein samples were collected and incubated with either anti-C/EBPα antibody (Cell Signaling, 8178, 1:50) or control nonimmune IgG. Immunocomplexes were then purified with protein G magnetic beads (Cell Signaling, 9006), washed, and resuspended in 2x Laemmli buffer. Western blot analysis was performed as described above using anti-C/EBPα antibody (Cell Signaling, 8178, 1:1000) and anti-NF-κB p65 antibody (Cell Signaling, 8242, 1:1000). As mouse IgG light chain has similar molecular weight with C/EBPα p42, C/EBPα p30 was subjected to band intensity analysis using Image Lab Software.

### Immunocytochemistry

Cells were fixed and permeabilized for 1 min using 0.1% Triton x100 (Thermo Fisher Scientific). After wash, cells were blocked with 5% goat serum for 1 h at RT and incubated with anti-NF-κB p65 antibody (Cell Signaling, 6956, 1:400) diluted in 1% goat serum overnight at 4 °C. Secondary goat anti-mouse antibody conjugated with Alexa Fluor488 (RRID: AB 2630356) was diluted at 1:500. Fluoroshield Mounting Medium with DAPI (Abcam, ab104139) was used to counterstain. Images were taken by Leica Stellaris 5 WLL Laser Scanning Confocal Microscopy and analyzed by ImageJ.

### ROS detection

C3H10T1/2 cells were seeded in 12-well plates and induced in high-glucose DMEM as previously described. At D4 or D7 after induction, cells were treated with 50 μg/ml ascorbate for 72 h. At D7 or D10 after induction, cells were stained with 5 μM CellROX Orange (Invitrogen, C10443) for 30 min. Images were taken and analyzed by Celigo Image Cytometer (Nexcelom Bioscience).

### Statistics

At least three independent experiments were performed for each panel of the figures. A two-tailed unpaired *t* test was used for analysis when two groups were compared, and sample data were normally distributed. When more than two groups were compared, we performed one-way ANOVA with either Tukey or Dunnett test based on normal spread of the data. With a significance level at 5%, we calculated mean values and standard deviations using GraphPad Prism software.

## Data availability

All data are contained within the article or supporting information.

## Supporting information

This article contains supporting information.

## Conflict of interest

The authors declare that they have no conflicts of interest with the contents of this article.
