# Supplementary material for: Mitochondrial permeability transition regulator, cyclophilin D, is transcriptionally activated by C/EBP during adipogenesis
Source: J Biol Chem. 2023 Nov 8;299(12):105458. doi: 10.1016/j.jbc.2023.105458 (PMC10716586; doi:10.1016/j.jbc.2023.105458)
Supplement: Supporting Figures S1–S6 and Table S1 [file mmc1.pdf]

Supporting Information for

Mitochondrial Permeability Transition Regulator, Cyclophilin D, is Transcriptionally Activated by C/EBP During Adipogenesis

Chen Yu[12], Rubens Sautchuk Jr[1], John Martinez[3], Roman A Eliseev[124].

1 Center for Musculoskeletal Research, University of Rochester, Rochester, NY 14624, USA
2 Department of Pathology, University of Rochester, Rochester, NY 14624, USA
3 Department of Biology, University of Rochester, Rochester, NY 14624, USA
4 Department of Pharmacology & Physiology, University of Rochester, Rochester, NY 14624, USA

Corresponding author:
Roman A. Eliseev
Email: roman_eliseev@urmc.rochester.edu

This PDF file includes:
Figures S1 to S6
Table S1

Figure S1

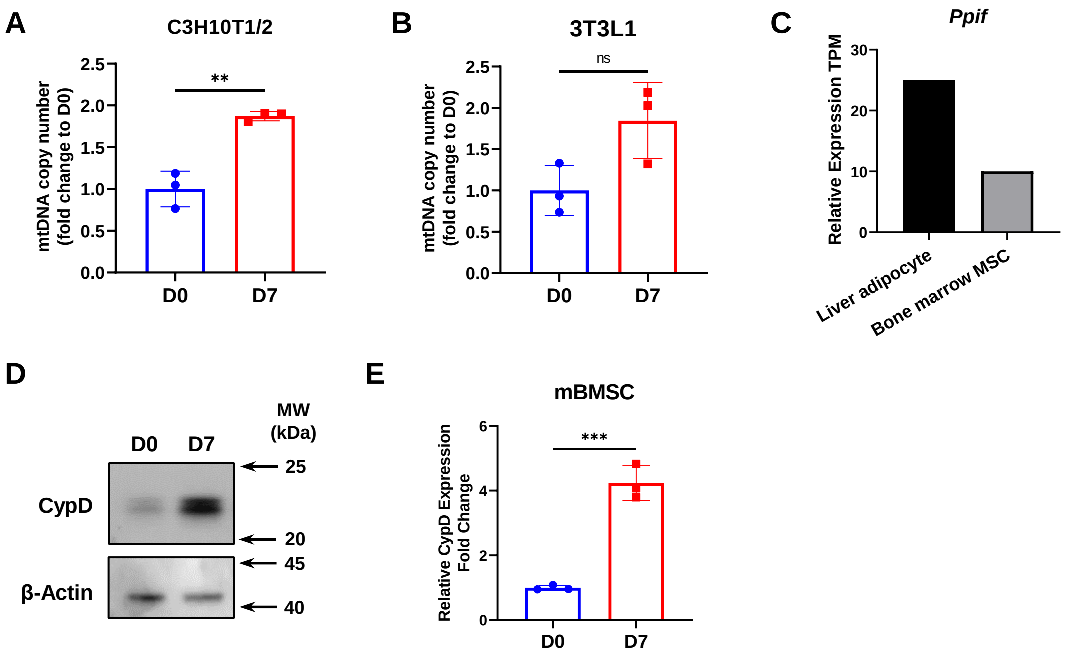

Supplementary Fig.1 CypD expression is upregulated during adipogenesis.
A) and B) Real-Time RT-PCR analysis of *mt-Co3* gene copy number was normalized to *18S* rRNA. C) Publicly available RNA-Seq data of *Ppif* expression in mouse. (https://fantom.gsc.riken.jp/data/). D) Representative western blot image of CypD protein expression in mBMSCs. E) Quantification of western blot analysis. Data are mean±SD (n=3), unpaired *t*-test. *p<0.05; **p<0.01; ***p<0.001.

Figure S2

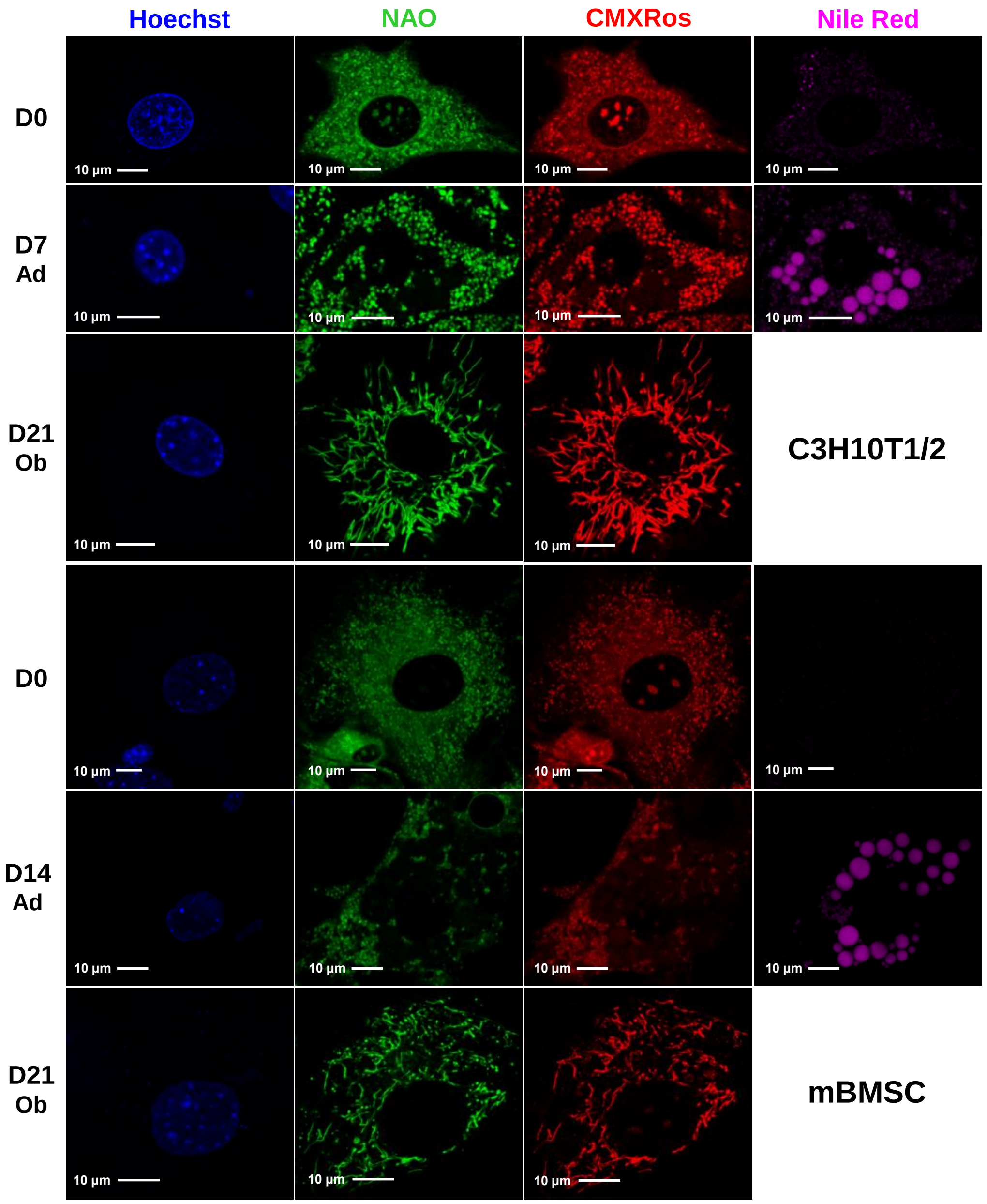

Supplementary Fig. 2 Representative images of mitochondrial morphology in each channel.
Confocal images of C3H10T1/2 (Fig. 3A) and mBMSCs (Fig. 3B) are displayed as individual channels. Blue channel: Hoechst; Green channel: NAO; Red channel: CMXRos; Magenta channel: Nile Red. Images were take by confocal microscopy at 63x magnification.

Figure S3

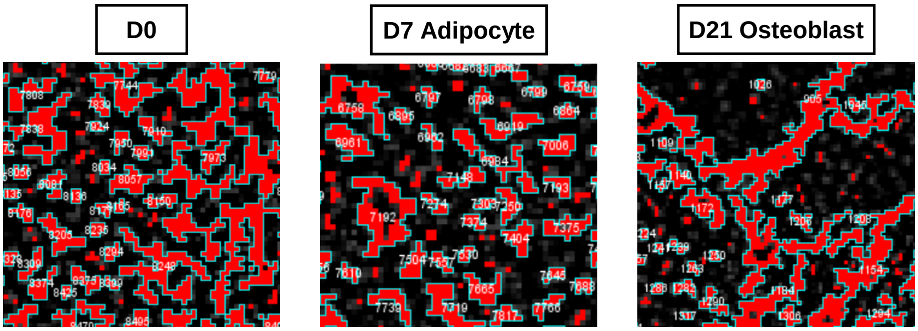

Supplementary Fig. 3 Representative images of mitochondrial morphology analysis in C3H10T1/2. Analysis was performed with CMXRos staining using ImageJ.

Figure S4

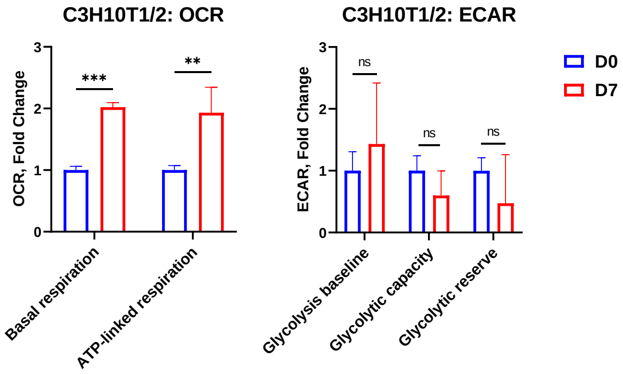

Supplementary Fig. 4 C3H10T1/2 cells activate mitochondrial function and maintain glycolysis level during osteogenesis.
C3H10T1/2 cells were cultured in Seahorse XFe96 plates and incubated in osteogenic media for 7 days. Oxygen consumption rate (OCR) and extracellular acidification rate (ECAR) were measured in an Agilent Seahorse XFe96 Analyzer. OCR and ECAR levels were normalized to cell number. OCR and ECAR values were quantified as fold change to D0. Data are mean±SD (n=4), unpaired *t*-test. *p<0.05; **p<0.01; ***p<0.001.

Figure S5

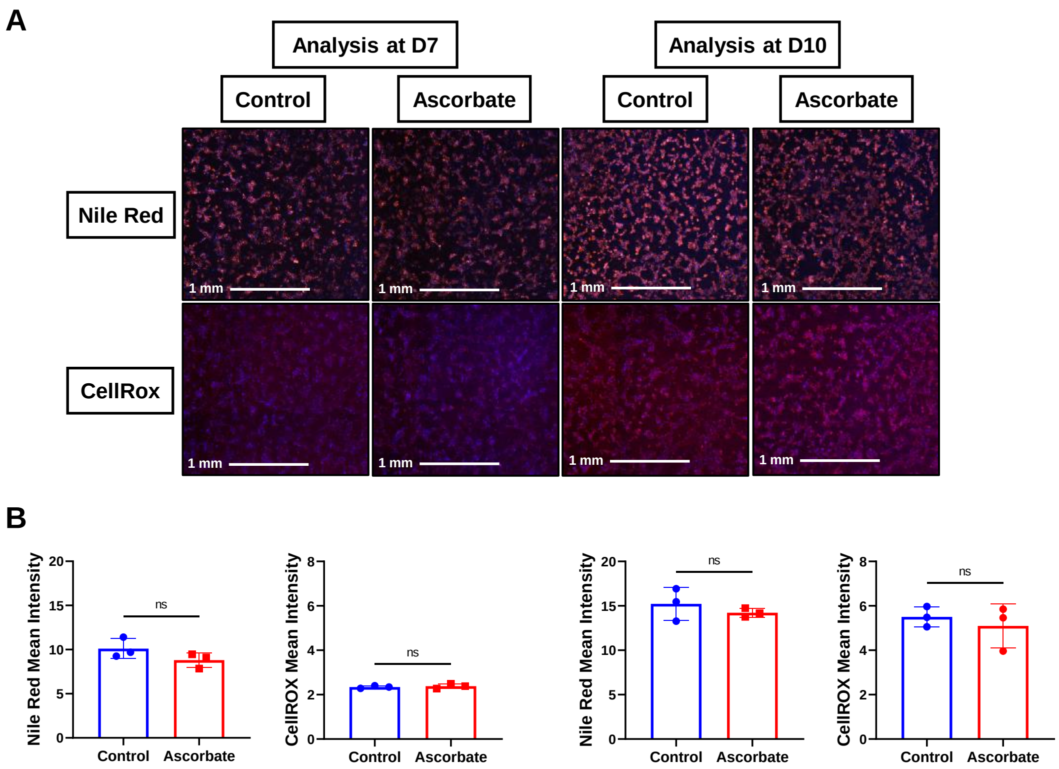

Supplementary Fig. 5 Effect of 72h Ascorbate treatment on adipogenesis in C3H10T1/2 cells.
C3H10T1/2 cells cultured in adipogenic media and treated with 50 µg/mL ascorbate for 72h either at D4 or at D7. A) representative images of Nile Red staining and CellROX Orange staining. B) Quantification of Nile Red staining and CellRox Orange staining. Data are mean±SD (n=3), unpaired *t*-test. *p<0.05; **p<0.01; ***p<0.001.

Figure S6

A

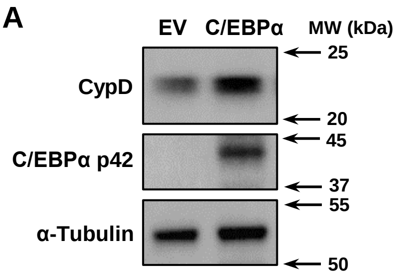

B

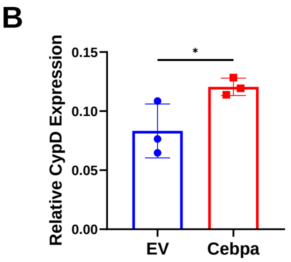

C

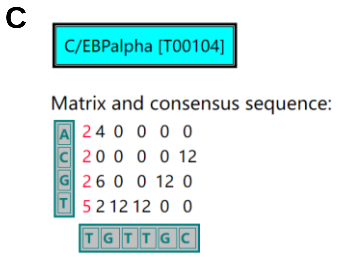

D

Mus musculus strain C57BL/6J chromosome 14, GRCm39
NCBI Reference Sequence: NC_000080.7
Upstream promoter -1.1kb- Chr14: 25693546 - 25694655

C/EBPα binding site

CACTGGAGATTCCCCGCTATGCTCTGAGCACACAGTCTAGGCTATGCCCATCGCTGTTGAACACAGAA
CAGCATTTAACAGTTAACAAATGCTTCTCTCTCTTGTTTGTTTATCCTCAGAACAGGTATGAGTAGAAAGC
AGGGTAATTTCTCATCGCTTCCTTGGTTGCAGGACTGGCTGAATCCAGCAGTAGGGAACCAGGGTTTT
CTAGCCCTAGTTAGAGCCTTTTTCTCTTTCTGAGCCTCAGTTTCCTAACTGTGTTGGCTCACATGCTCTC
GCTGGCTGGCATTCATTTGATTCCCAGGCTGGTGCTGTGCCCAGTGCACCAGGGTTTAAAGTCCTGCC
TGTGGCTCTTCTGCAGGCTCCTTTCCTTTTATATCACACACTGCCAACTTCCTGGCATCCCTAGAGAATG
GTTGCTTAGGAAGTTACCTGCAGGTGTGGTGCAGTTGGCAAGGAAGTTTATTCCAGGTGAATGTAAATC
TAGGGGGCATTCCAGGGGGTGTAAATCTAGCCCCGCACTCTGCTGGCCTGGCTGGAGGTGGTGGGAA
CAGACAAGGGCTGTCAGAGTGGGCTGGGACTGAGGCCTGGCAGATGCACTATTGGTTCGGCAAACTG
TATTTTCAGTCCTGGCGGAGGATCTGGTCTCTAGAAGCAAAAGGAGACAAACTCAGCCCTGGAGCCGG
CAGCCTGAGGTCACCTTGTGGGTGTCTTAGACTGTATCTCTGCTTCTCTGTTCTTTCTGTTATCTCTCCC
TTTCTGAGCCACCTCAGATTCCCCCACCTACACCCCCCCACGTCTTTCTGAATCTCAGGATCAACCTTT
CTCTTGATCTAGAAGCACTTTCAAAGGACTTCCGGCTCAGTGTGTAGGGATGGAGGGCGGGGCACAAC
GCACGAGCCGATTACGCTGCCAGCGGCAACCGGCAGGGGACGCTAGCGAACAGGTTCCGAGGGGGC
GCGGCGGAGGCGTGGAGGGGGGCGTGGTCTCGAAGCCAGCCGACCAATAAAGGCGGCGGCGCGCG
GCCTAGGTATCGCTTCCTGGGTCGTACGACCTTGCCTGTGTCTGCTCTGAGTTCTTCCGCGCGCCCTC
GCCCGACCCGCGACAGCGATG→

Position 2-4: CGC

mutated C/EBPα binding sites

CACTGGAGATTCCCCGCTATGCTCTGAGCACACAGTCTAGGCTATGCCCATCGCTGTTGAACACAGAA
CAGCACGCAACAGTTAACGCATGCTTCTCTCTCTTGTTTGTTTATCCTCAGAACAGGTATGAGTAGAAAG
CAGGGTAACGCCTCATCGCTTCCTTGGTTGCAGGACTGGCTGAATCCAGCAGTAGGGAACCAGGGTTT
TCTAGCCCTAGTTAGAGCCTTTTTCTCTTTCTGAGCCTCAGTTTCCTAACTGTGTTGGCTCACATGCTCT
CGCTGGCTGGCATTCACGCGATTCCCAGGCTGGTGCTGTGCCCAGTGCACCAGGGTTTAAAGTCCTG
CCTGTGGCTCTTCTGCAGGCTCCTTTCCTTTTATATCACACACTGCCAACTTCCTGGCATCCCTAGAGAA
TGGTTGCTTAGGAAGTTACCTGCAGGTGTGGTGCAGTTGGCAAGGAAGTTTATTCCAGGTGAATGCGC
ATCTAGGGGGCATTCCAGGGGGTGCGCATCTAGCCCCGCACTCTGCTGGCCTGGCTGGAGGTGGTG
GGAACAGACAAGGGCTGTCAGAGTGGGCTGGGACTGAGGCCTGGCAGATGCACTATTGGTTCGGCAA
ACTGTACGCTCAGTCCTGGCGGAGGATCTGGTCTCTAGAAGCAAAAGGAGACAAACTCAGCCCTGGA
GCCGGCAGCCTGAGGTCACCTTGTGGGTGTCTTAGACTGTATCTCTGCTTCTCTGTTCTTTCTGTTATC
TCTCCCTTTCTGAGCCACCTCAGATTCCCCCACCTACACCCCCCCACGTCTTTCTGAATCTCAGGATCA
ACCTTTCTCTTGATCTAGAAGCACTTTCAAAGGACTTCCGGCTCAGTGTGTAGGGATGGAGGGCGGGG
CACAACGCACGAGCCGATTACGCTGCCAGCGGCAACCGGCAGGGGACGCTAGCGAACAGGTTCCGA
GGGGGCGCGGCGGAGGCGTGGAGGGGGGCGTGGTCTCGAAGCCAGCCGACCAATAAAGGCGGCG
GCGCGCGGCCTAGGTATCGCTTCCTGGGTCGTACGACCTTGCCTGTGTCTGCTCTGAGTTCTTCCGCG
CGCCCTCGCCCGACCCGCGACAGCGATG→

Supplementary Fig. 6 C/EBPα overexpression and mutagenesis experiment in C3H10T1/2 cells.
A) Representative image of CypD, C/EBPα and α-Tubulin expression. C3H10T1/2 cells were transfected with 0.4 μg pCMV-C/EBPα vector or empty vector (EV) as control. B) Quantification of western blot analysis. C) Predicted C/EBPα binding motif by PROMO web-based tool. D) Predicted C/EBPα binding sites and mutated binding sites in mouse 1.1kb *Ppif* promoter region. Data are mean±SD (n=3), unpaired *t*-test. *p<0.05; **p<0.01; ***p<0.001.

Table S1

Primer	Sequence 5’-3’
Mouse *B2m*_forward	AATGGGAAGCCGAACATAC
Mouse *B2m*_reverse	CCATACTGGCATGCTTAACT
Mouse *Adipoq*_forward	GAGAAGGGAGAGAAAGGAGATG
Mouse *Adipoq*_reverse	TGAGCGATACACATAAGCGG
Mouse *Cebpa*_forward	ATAAGAACAGCAACGAGTACC
Mouse *Cebpa*_reverse	GCGGTCATTGTCACTGGTC
Mouse *Pparg*_forward	GGCCTCCCTGATGAATAAAG
Mouse *Pparg*_reverse	GCCAAGTCACTGTCATCTAAT
Mouse *Ppif*_forward	CATGTACCC GAACAGAAC
Mouse *Ppif*_reverse	CATGTACCC GAACAGAAC
Mouse *Rela*_forward	TGAGTCAGATCAGCTCCTAAG
Mouse *Rela*_reverse	CCTCCGAAAGCGAGATAAAG
Mouse *mt-Co3*_forward	CGAAACCACATAAATCAAGCCC
Mouse *mt-Co3*_reverse	CTCTCTTCTGGGTTTATTCAGA
Mouse *18s*_forward	TAGAGGGACAAG TGGCGTTC
Mouse *18s*_reverse	CGCTGAGCCAGTCAGTGT
Distal *Ppif* promoter_forward	AAGACTCGAGTGGAGATTCCCCGCTAT
Distal *Ppif* promoter_reverse	GGTAATTTCTCATCGCTTCCTTGAAGCTTAAGA

Table S1. Primer sequences used for real-time RT-PCR and standard PCR.
